# Detection of fraudulent financial statements using the hybrid data mining approach

**DOI:** 10.1186/s40064-016-1707-6

**Published:** 2016-01-27

**Authors:** Suduan Chen

**Affiliations:** Department of Accounting Information, National Taipei University of Business, No. 321, Sec. 1, Jinan Rd., ZhongZheng District, Taipei, 100 Taiwan

**Keywords:** Fraudulent financial statements, Decision tree CART, Decision tree CHAID, Bayesian belief network, Support vector machine, Artificial neural network

## Abstract

The purpose of this study is to construct a valid and rigorous fraudulent financial statement detection model. The research objects are companies which experienced both fraudulent and non-fraudulent financial statements between the years 2002 and 2013. In the first stage, two decision tree algorithms, including the classification and regression trees (CART) and the Chi squared automatic interaction detector (CHAID) are applied in the selection of major variables. The second stage combines CART, CHAID, Bayesian belief network, support vector machine and artificial neural network in order to construct fraudulent financial statement detection models. According to the results, the detection performance of the CHAID–CART model is the most effective, with an overall accuracy of 87.97 % (the FFS detection accuracy is 92.69 %).

## Background

Financial statements are a company’s basic documents that reflect its financial status (Beaver 1966; Ravisankar et al. 2011). The financial statement is the main basis for decision -making on the part of a vast number of investors, creditors and other persons in need of accounting information, as well as a concrete expression of business performance, financial status and the social responsibility of listed companies and OTC companies. However, in recent years, cases of fraudulent financial statements have become increasingly serious (Wells 1997; Spathis et al. 2002; Kirkos et al. 2007; Yeh et al. 2010; Humpherys et al. 2011; Kamarudin et al. 2012). Since the Asian Financial Crisis in 1997, there have been many cases of fraudulent financial statements in Taiwan and the United States. Examples include the Enron case in 2001, the WorldCom case in 2003 in the United States, and the ABIT Computer, Procomp, Infodisc and Summit Technology cases in 2004 in Taiwan. Given these incidents, it has become important to be able to detect fraudulent behavior prior to its occurrence.

Data mining is a key tool for dealing with complex data analysis and classification. It identifies valuable events that are hidden in large amounts of data for analysis, and summarizes the data in a structured model to provide a reference for decision-making. Data mining has many different functions, such as classification, association, clustering and forecasting (Seifert 2004). The classification function is used most frequently. The classification results can be used as the basis for decision-making and for prediction purposes.

Fraudulent financial statements can be viewed as a typical classification problem (Kirkos et al. 2007). The classification problem involves performing computation using the variable characteristics of some known classification data, in order to obtain classification-related classification rules. Subsequently, the unknown classification data are inputted into the rules in order to obtain the final classification results. Regarding the issue of fraudulent financial statements, much of the past research has proposed the use of the data mining method because of its superiority in terms of forecasting after inputting large amounts of data for machine learning, as well as its accuracy in terms of classification and forecasting, which is far higher than that of conventional regression analysis. For example, artificial neural network (ANN), decision tree (DT), Bayesian belief network (BBN), and support vector machine (SVM) methods have been applied in order to detect fraudulent financial statements.

It is therefore urgent that we establish an effective and accurate fraudulent financial statement detection model, because conventional statistical models experience great disadvantages in detecting fraudulent financial statements due to their relatively high error rate. Some scholars have proposed using data mining techniques in order to judge concerns about business operational continuity and thereby reduce judgment errors. However, prior studies are neither sufficient nor complete. For example, most use only 1–2 statistical methods, without model comparison, Furthermore, most use a one-stage statistical treatment in order to establish the detection model, which is not prudent. The main purpose of this study is to propose a better model to detect potentially fraudulent financial statements, so that the losses incurred by investors and caused by auditors can be reduced. Compared with previous literature, this study adopts: (1) a two-stage statistical treatment; (2) five data mining techniques to create the detection model for comparison of model accuracy; (3) tenfold cross validation which is thought to be prudent and is commonly used in the academic field. In short, this study is not only prudent, but is also innovative and makes significant contributions to the literature. This study selects the major variables by applying the DT techniques of the Chi squared automatic interaction detector (CHAID) and classification and regression trees (CART). Moreover, this study establishes classification models for comparison by combining CART, CHAID, BBN, SVM, and ANN data mining techniques.

## Fraudulent financial statements

### Reviews on fraud

Regarding the processing of financial statements, Enron applied the highly controversial Special Purpose Entities account in order to solve the problem of financing liabilities. However, the company did not have to list its increased financing liabilities in the balance sheet, which is known as off-balance sheet financing in accounting. The US Committee of Sponsoring Organizations of the Treadway Commission (COSO) (Beasley et al. 1999) and SAS No. 99, 2002 defined a fraudulent financial statement as either intentional or reckless conduct based on false information or omissions that results in significantly misleading financial reports. The cost of the prevention of fraudulent financial statements in the United States is estimated to be in the billions of dollars each year (Humpherys et al. 2011). The US Association of Certified Fraud Examiners (ACFE) classifies fraud into six types: (1) providing false financial information; (2) misuse or misappropriation of corporate assets; (3) improper support or loans; (4) improperly acquiring assets or income; (5) improper circumvention of costs or fees; and (6) improper manipulation of financing by executives or board members. The Taiwan Accounting Research and Development Foundation released the Auditing Standards No. 43 communiqué in 2006, in which fraud was defined as the management, control unit or one or more employees deliberately using deception and other methods to acquire improper or illegal benefits. Therefore, it could be concluded that the four elements of fraud are: (1) serious erroneous expressions of the nature of transactions, (2) knowingly violating rules, (3) the victim accepting a misstatement as fact; and (4) damage due to financial losses caused by the above three situations. Misstatement fraud relating to financial statement auditing includes financial report fraud and misappropriation of assets. Financial reporting fraud refers to untrue financial statements which aim to deceive users. The US Security and Exchange Commission (SEC) state that financial statements should “provide a comprehensive overview of the company’s business and financial condition and include audited financial statements”.

Fraudulent financial statements are intentional and illegal acts that result in misleading financial statements or misleading financial disclosure (Beasley 1996; Rezaee 2005; Ravisankar et al. 2011). Stakeholders are adversely affected by misleading financial reports (Elliot and Willingham 1980). Most prior studies use conventional statistical multivariate analysis, notably logistic regression analysis (Beasley 1996; Summers and Sweeney 1998; Bell and Carcello 2000; Spathis et al. 2002; Sharma 2004; Uzun et al. 2004; Chen et al. 2006; Humpherys et al. 2011). Conventional statistical methods require compliance with the limitations of specific hypotheses, for example, the avoidance of collinearity of independent variables and the distribution of data (Chiu et al. 2002). However, according to Chen (2005), regarding variables, empirical financial variables often cannot comply with relevant statistical conditions, such as normal distribution. Therefore, the machine learning method, which requires no statistical hypotheses of data combinations, has emerged and been used by scholars as a classifier. The empirical results suggest that the machine learning method has a positive classification effect.

### Application of data mining in detecting fraudulent financial statements

Most previous research has used the conventional statistical method to make decisions regarding operational continuity. However, this method causes a number of disadvantages in terms of judgment and its error rate is relatively high. In recent years, some studies have applied data mining techniques in order to detect fraudulent financial statements and thereby reduce judgment errors. Studies applying DT techniques to detect fraudulent financial statements include: Hansen et al. (1992), Koh (2004), Kotsiantis et al. (2006), Kirkos et al. (2007), and Salehi and Fard (2013). Studies applying BBN techniques to detect fraudulent financial statements include: Kirkos et al. (2007), and Nguyen et al. (2008). Studies that apply SVM techniques to detect fraudulent financial statements include: Zhou and Kapoor (2011), Shin et al. (2005), Chen et al. (2006), Yeh et al. (2010), Ravisankar et al. (2011), Pai et al. (2011). Studies applying ANN techniques to detect fraudulent financial statements include: Hansen et al. (1992), Coats and Fant (1993), Fanning and Cogger (1998), Koh (2004), Chen et al. (2006), Kirkos et al. (2007), Ravisankar et al. (2011), and Zhou and Kapoor (2011). The judgment accuracy rates of using data mining techniques to detect fraudulent financial statements vary, and the construction of the model is neither complete nor perfect. As stated above, most studies only use 1–2 data mining techniques, without offering model comparison; and most use one-stage statistical treatment to establish the detection model, which is not prudent.

Prior studies point out that using data mining techniques to detect fraudulent financial statements is superior to adopting a conventional regression analysis in terms of accuracy. This study proposes using a two-stage fraudulent financial statement detection model using the DT CART and CHAID algorithms in variable selection in order to identify influential variables. Next, this study applies CART, CHAID, BBN, SVM and ANN in order to construct the fraud detection model and conducts a pairwise comparison of the testing groups of each model in terms of classification accuracy, Type I errors, and Type II errors to identify the model with the optimal accuracy.

## Methods

This study utilizes several data mining techniques: DT, BBN, SVM, and ANN.

### Decision tree

**Fig. 1:**
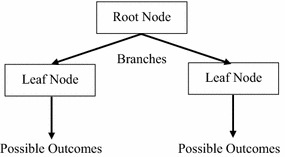
DT concept diagram

DT is the simplest inductive learning method (Arminger et al. 1997). As a data mining technique, it is able to handle continuous and non-continuous variables (DT concept diagram is shown as Fig. 1). The decision-making processes of the judgment tree method are as follows: (1) draw tree diagram; (2) mark various status probabilities and income and loss values on the probability branch; (3) calculate the expectancy value of each plan and mark it on the corresponding status node of that plan; (4) trim the branches, compare the expectancy value of each plan and mark it on the plan branch and trim the small expectancy value; (5) the remaining plan is the best one. Establishing the tree diagram and summarizing the rules primarily depends on classified and known facts. These rules are mutually exclusive, and the generated DT can make out-of-sample predictions. The most commonly used DT algorithms include CART, CHAID and C5.0 (Viaene et al. 2005). C5.0 (Quinlan 1993) is developed from ID3 (Quinlan 1986a, b). Since ID3 has limitations in terms of use and cannot process continuous numerical data, Quinlan improved it and developed C5.0 to enable it to process continuous and non-continuous values. The difference between CHAID and CART, C5.0 is that CHAID is only limited to the processing of category variables, in case of continuous variables, an interval must be used to convert it into other variables. Another difference relates to trimming; CART, C5.0 is the first method to mechanically use data training excessively and then trim, but CHAID is used to stop the spreading of branches before excessive mechanical use.

DT C5.0 is primarily divided into two parts. The first is the classification criteria. The DT is constructed based on the computation of the gain ratio. The information gained from Eq. (1) is used to compute the data set profits before and after the test. As in Eq. (2), it is defined as the subtraction of the pre-test information from post-test information. Entropy in Eq. (1) is used in order to compute the impurity of the computation data set, herein referred to as the chaos degree. When the degree of chaos of the data set reaches the highest level, the value will be 1. Therefore, when the chaos degree of the post-test data set is lower, the information gain will be greater and is more beneficial to the construction of the DT.

1 $$Gain\,Ratio(S,A) = \frac{Information\,Gain(S,A)}{Entropy(S,A)}$$

2 $$Gain(S,A) = Entropy(S) - \sum\limits_{v \in values(A)} {\frac{{\left| {S_{v} } \right|}}{|S|}} Entropy(S_{v} )$$

Values (A) is the set of all possible values of attribute A, S_v_ is the subset of values v of attribute A in S. The first item of this equity is the entropy of the original set; and the second item is the expectancy value of S after classification with A. The expected entropy described in the second item is the weighted sum of each subset, and the weighted value is the proportion |S_v_|/|S| of samples belonging to S_v_ in the original sample S.

The second part is the pruning criteria, which uses error based pruning (EBP) to carry out the appropriate pruning of the DT and thereby improve the classification accuracy rate. EBP is from pessimistic error pruning (PEP), both are proposed by Quinlan. The most important feature of EBP is its ability to make judgments according to the error rate. It computes the error rate of each node and determines the nodes that cause a rise in the error rate of the DT before engaging in appropriate pruning of these nodes to improve the accuracy rate of the DT.

### Bayesian belief network

BBN, first proposed by Pearl (1986), plays an important role in issues of uncertainty and inference, and has been extensively applied in many cases, such as natural resources (Newton et al. 2007), and medical diagnosis and software cost evaluation (Stamelos et al. 2003). Its inference depends on the acquisition of new information. According to the Bayes’ theorem, the probability values of the status of relevant nodes are adjusted. It is a good method for establishing the model and is able to reflect the uncertain factors of reality. The structure of the diagram presents causal relationships and infers final results by the computation of probability. When given new information, BBN is able to regulate probability (Tang et al. 2007). Namely, when the probability value is adjusted, all the relevant nodes on the network can be adjusted according to the conditional probabilities.

**Fig. 2:**
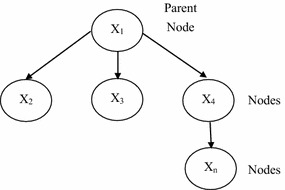
BBN concept diagram

BBN is a directed acyclic graph consisting of a series of nodes and arrows in between nodes, and includes a number of decision-making variables (a BBN concept diagram is shown as Fig. 2). These decision-making variables are connected in the same direction to form the parent-offspring relationship. The nodes represent the decision-making variables and the arrows represent the dependency relationship between various variables. These variables can be discrete or continuous (Tang et al. 2007). If the arrow direction is from A to B, this means that B originates from A, and that A is the parent node and B is the offspring node. The arrows represent the causal relationship and strength. If each node x contains different parent nodes (Parents(x)), then the conditional probability of getting all parent nodes and node x is as shown in Eq. (3), hence the conditional probability table for node x can thus be developed. The probability combinations of n attributes $({\text{x}}_{1} ,{\text{x}}_{2} , \ldots ,{\text{x}}_{\text{n}})$ are as shown in Eq. (4)

3 $$P\left( {x\left| {Parents(x)} \right.} \right)$$

4 $$P(x) = P(x_{1} ,x_{2} , \ldots ,x_{n} )\prod\limits_{i = 1}^{n} {P\left( {x_{i} \left| {P_{ai} } \right.} \right)}$$

### Support vector machine

SVM is a set of artificial intelligent learning methods proposed by Vapnik (1995). It is a machine learning method based on statistical learning theory and SRM (structural risk minimization). It primarily depends on using input training data to find an optimal separating hyperplane that can distinguish two or more types (class) of data through the learning mechanism. It is a supervised learning, predication and classification method for data mining.

**Fig. 3:**
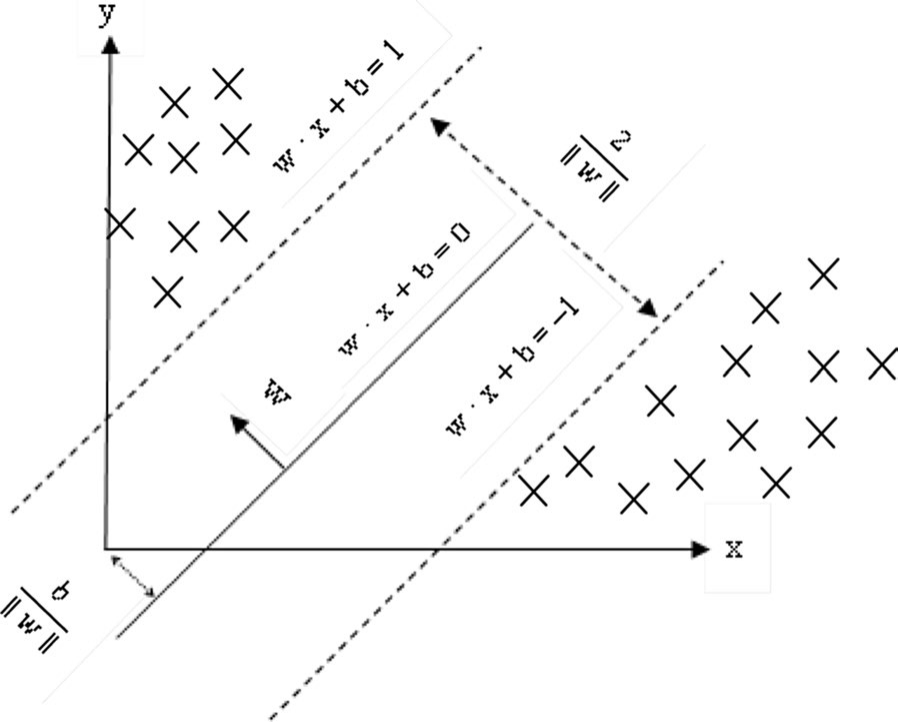
SVM concept diagram

The SVM concept diagram is shown in Fig. 3. SVM processing can be classified into linear divisibility and linear non-divisibility.

### Linear divisibility

Let the training set be $(x_{1} ,y_{1} ), \ldots ,(x_{l} ,y_{l} )$, $x \in R^{n} ,\,y \in \{ 1, - 1\} ,$ in which x is the input variable, and the data will classify a super-plane into two categories, one is 1, and the other is −1. If such data can be distinguished correctly, and the nearest vector of each classification is at a maximum distance from the super plane, then the super plane is most suitable for divisibility. We express the super plane with the following formula:

5 $$y_{i} (w,x) + b \ge 1,\quad i = 1, \ldots ,l$$

The maximum spacing of the two nearest vectors of Eq. (5) is as shown in Eq. (6):

6 $$\begin{aligned} p(w,b) & = \mathop {\hbox{min} }\limits_{{x_{i} ,y_{i} = - 1}} d(w,b:x_{i} ) + \mathop {\hbox{min} }\limits_{{x_{i} ,y_{i} = 1}} d(w,b:x_{i} ) \\ & = \mathop {\hbox{min} }\limits_{{x_{i} ,y_{i} = - 1}} \frac{{\left| {(w,x_{i)} + b} \right|}}{\left\| w \right\|} + \mathop {\hbox{min} }\limits_{{x_{i} ,y_{i} = 1}} \frac{{\left| {(w,x_{i)} + b} \right|}}{\left\| w \right\|} = \frac{2}{\left\| w \right\|} \\ \end{aligned}$$

The maximum distance (6) can be expressed as minimum $\Phi (w) = \frac{1}{2}\left\| w \right\|^{2} ,$ Lagrange relaxation is used to calculate the super plane most suitable for divisibility, as in Eq. (7):

7 $$w^{*} = \sum\limits_{i = 1}^{l} {a_{i} y_{i} x_{i} } \quad b^{*} = - \frac{1}{2}\left( {w^{*} ,x_{r} + x_{s} } \right)$$

where *x*_*r*_, *x*_*s*_ are any support vectors that each classification can meet $\alpha_{r} ,\,\alpha_{s} > 0,\,y_{r} = - 1,\,y_{s} = 1.$ We acquire the clear hard classifier, as in Eq. (8):

8 $$f(x) = \text{sgn} \left( {\left( {w^{*} ,x} \right) + b} \right)$$

If the condition of incapable or complete classification is not considered, the soft classifier is as shown in Eq. (9):

9 $$f(x) = h\left( {w^{*} ,x} \right) + b\quad where \quad h(z) = \left\{ {\begin{array}{*{20}l} { - 1{:}\quad z < - 1} \hfill \\ {z{:}\quad - 1 \le z \le 1} \hfill \\ { + 1{:}\quad z > 1} \hfill \\ \end{array} } \right.$$

### Linear non-divisibility

Vapnik (1995) imports the concept of the cost function as related to wrong classifications in order to calculate the super plane most suitable for divisibility, which can be expressed as Eq. (10):

10 $$\begin{aligned} & \hbox{min} \Phi \left( {w,\xi } \right) = \frac{1}{2}\left\| w \right\|^{2} + C\sum\limits_{i = 1}^{l} {\xi_{i} } \\ & s.t.\quad y_{i} [(w,x) + b] \ge 1 - \xi_{i} ,\quad i = 1, \ldots ,l. \\ & \quad \quad where\quad \xi \ge 0 \\ \end{aligned}$$

where $\xi_{i}$ is the error item of wrong classification, C is a given parameter value, and Lagrange relaxation can be obtained, as in Eq. (11) (Ribeiro et al. 1989; Larsson et al. 1996):

11 $$\Phi (w,\xi ) = \frac{1}{2}\left\| w \right\|^{2} + C\sum\limits_{i = 1}^{l} {\xi_{i} - \sum\limits_{i = 1}^{l} {\alpha_{i} \left( {y_{i} \left[ {w^{T} x_{i} + b} \right] - 1 + \xi_{i} } \right) - \sum\limits_{i = 1}^{l} {\beta_{i} \xi_{i} } } }$$

where *α*, *β* are Lagrange constants.

### Artificial neural network

**Fig. 4:**
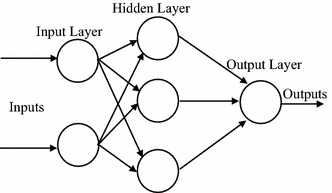
ANN concept diagram

ANN is a system that imitates a biological neural network’s computational capabilities (the ANN concept diagram is shown in Fig. 4) because biological vision and hearing capabilities are superior to computer systems at the time. Therefore, it is expected to gain powerful computational capabilities by imitation. In the 1940s, scientists imitated the simplest neural cell modes in order to establish the most primitive ANN. McCulloch and Pitts (1943) propose a simplified neural cell mathematical model in order to simulate the human brain’s computational capabilities.

**Fig. 5:**
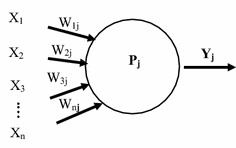
ANN neural cell

The neural cell is the most basic unit of ANN. Assuming there is a neural cell, j, which obtains an input variable, x, respectively, from the n neural cells in the upper layer; each input variable has a linkage weight, w. The neural cell combines all the input variables according to their weights into the combination function. The neural cell’s activation function reuses the combination function to convert it into the output signal. The theory of ANN neuron is represented in Fig. 5, in which n represents the number of input variables, Xi is the ith input variable, Wij is the weight of the ith variable of the jth neural cell, and Pj is the combination function of the jth neural cell. If represented by activation function f(x), then Yj = f (Pj) is the output value of the jth neural cell.

In the evolutionary process of neural networks, it is necessary to have a parameter with a training model in order to train the required weight for the forecast variable at the outset, and such parameters are randomly generated. Given this, the parameters used in each training model differ. Finally, the weights of forecast variables generated by the neural network are also different, but the error will meet a minimal value, so the neural network is a deduction acquired through a trial-and-error method, and its purpose is to minimize errors in the model forecast results. For the same data, the weighs trained are not equal, and so the essence of a neural network is to emphasize the training model. Therefore, there will be no judgment formula for the result of the neural network model, rather only the judgment result is obtained.

### Sample and variable selection

#### Data sources

This study investigates Taiwan’s listed and OTC companies who released fraudulent financial statements during the period of 2002–2013. From companies listed in the Securities and Futures Investors Protection Center, and among the major securities criminal prosecution and judgment publications by the Securities and Futures Bureau, from those companies that violated the term of “misrepresented expression of financial statements”, Articles 155 and 157 of the Securities Transaction Act, and No. 43 Bulletin of Auditing Standards, this study selected 44 fraudulent companies. Those companies included one in the building and construction industry, two in the food processing industry, two in the textile and fiber industry, seven in the semiconductor industry, nine in the electronics industry, four in the photoelectric industry, one in the telecommunications industry, six in other electronics industries, two in the steel industry, one in the rubber industry, one in the shipping industry, three in the software services industry, two in the electric-mechanical industry, one in the electric appliance and cable industry, and two in other industries. The financial statements of the financial industry are not comparable to other industries and the financial ratio is different from that of general industries, and thus the financial industry was eliminated.

In order to control numerous external environmental factors such as time, industry, and company size, the matching method can be adopted. Hence, this study adopted the matching sample design concept proposed by Kotsiantis et al. (2006) in order to match one fraudulent company with three normal companies. This study selects normal companies with similar total assets in the same industry in the previous year of the fraudulent financial statements as the matching sample. A total of 176 companies are selected, including 44 fraudulent companies and 132 normal companies who have not engaged in fraudulent behavior.

#### Variable definition

The dependent variable is used to classify companies into normal companies, represented by 0, and fraudulent companies, represented by 1. This study uses 30 independent variables including 23 financial variables and seven non-financial variables. The research variables and definitions are shown in Table 1.

**Table 1:** Research variables and definitions

Variables	No.	Variable description	Definition/formula (the year before the year of fraud)
Financial variables	X 1	Accounts receivable ratio	Accounts receivable ÷ total assets
	X 2	Current assets ratio	Current assets ÷ total assets
	X 3	Fixed assets ratio	Fixed assets ÷ total assets
	X 4	Operating income to total assets	Operating income ÷ total assets
	X 5	Net income to total assets	Net income ÷ total assets
	X 6	Net income to fixed assets	Net income ÷ fixed assets
	X 7	The proportion of cash against total assets	Cash ÷ total assets
	X 8	Natural logarithm of total assets	ln total assets
	X 9	Natural logarithm of total liabilities	ln total liabilities
	X 10	Gross profit ratio	Gross profit ÷ net sales
	X 11	Operating expenses ratio	Operating expenses ÷ net sales
	X 12	Debt ratio	Total liabilities ÷ total assets
	X 13	Current ratio	Current assets ÷ current liabilities
	X 14	Quick ratio	Quick assets ÷ current liabilities
	X 15	Inventory turnover	Cost of goods sold ÷ average inventory
	X 16	Cash flow ratio	Operating cash flow ÷ current liabilities
	X 17	Pre-tax profit ratio	Pre-tax profit ÷ net sales
	X 18	Accounts receivable turnover	Net sales ÷ average accounts receivable
	X 19	Sales growth rate	(Current year’s sales − last year’s sales) ÷ last year’s sales
	X 20	Debt-to-equity ratio	Total liabilities ÷ total equity
	X 21	Returns on assets before tax, interest, and depreciation	Income before tax, interest and depreciation ÷ average total assets
	X 22	The ratio of current liabilities against total assets	Current liabilities ÷ total assets
	X 23	Total asset turnover	Net sales ÷ average total assets
Non-financial variables	X 24	The major stockholders’ stockholding ratio	Number of stocks held by the major shareholders ÷ total number of common stocks outstanding
	X 25	Duality of board director and CEO	If duality of board director and CEO existed, it is set as 1; otherwise, 0
	X 26	Size of the board of directors	Number of directors
	X 27	The ratio of pledged stocks held by directors and supervisors	Number of pledged stocks held by directors and supervisors ÷ number of stocks held by directors and supervisors
	X 28	The ratio of stocks held by directors and supervisors	Number of stocks held by directors and supervisors ÷ total number of common stocks outstanding
	X 29	Audited by BIG4 (the big four CPA firms)	1 for companies audited by BIG4, otherwise, it is 0
	X 30	Number of outside supervisors	Number of outside supervisors

#### Research design

**Fig. 6:**
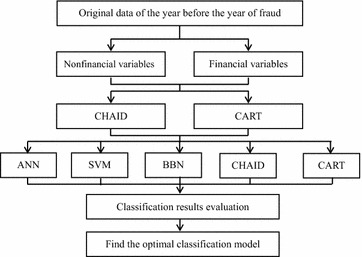
Research design and procedure

Previous research is susceptible to the following disadvantages: (1) Most only use 1–2 statistical methods without model comparison; (2) Most only use one-stage statistical treatment to establish the detection model, which is not prudent. Therefore, a two-stage statistical treatment is used in this research (data mining techniques are used), and the tenfold cross validation generally thought to be prudent in the academic field is also used. The accuracy of the model is also compared in order to establish a relatively prudent “Fraudulent Financial Statements” detection model. This study selects 30 variables that can affect fraudulent financial statements from the literature and applies the DT CART and CHAID algorithm in variable selection in order to identify influential variables. Next, CART, CHAID, BBN, SVM and ANN are applied in order to establish fraud warning models, and a pairwise comparison of the testing groups is conducted in terms of detection accuracy, Type I errors, and Type II errors. The research design and procedure are shown in Fig. 6.

## Results and discussion

This study selects 30 variables in order to determine the variables with the greatest impact on fraudulent financial statements. The selected variables are processed in the second stage using BBN, SVM and ANN modeling and classification performance tests.

### Variable selection

Since this study selects relatively more variables, DT is applied in order to identify the important and representative variables. In this study, SPSS Clementine is used as the software for DT variable selection, and CART and CHAID are used for variable selection.

### CART algorithm selection

The CART is a data mining algorithm developed by Breiman et al. (1984). It is a binary segmentation DT technique used for application with continuous or classified non-parameter data. Segmentation condition selection is determined by the data classes and their attributes. Segmentation conditions are determined by Gini rules. Segmentation divides the data into two subsets, and the conditions for the next segmentation are found in the subset.

Table 2 illustrates the results of using the CART algorithm in selection. Only 4 of the 30 variables were left after selection by the CART algorithm, including: X02 current assets ratio, X09 natural logarithm of total liabilities, X16 cash flow ratio, and X19 sales growth rate. The sequence in terms of the importance of the variables is X16, X02, X19 and X09.

**Table 2:** Selection results of decision tree CART

Variables	Variable importance
X16 (cash flow ratio)	0.496
X02 (current assets ratio)	0.480
X19 (sales growth rate)	0.016
X09 (natural logarithm of total liabilities)	0.008

### CHAID algorithm selection

The CHAID is a method that applies the Chi square test in the computation of the P-values of the broken nodes of the branches and leaves of the DT in order to determine whether or not segmentation should be continued. CHAID can prevent the excessive use of data and allow the DT to stop segmentation. In other words, CHAID is able to complete pruning before the establishment of the model.

Table 3 illustrates the results of applying the CHAID algorithm in selection. Only 6 of the 30 variables were left after the CHAID algorithm selection, including: X02 current assets ratio, X11 operating expenses ratio, X12 debt ratio, X14 quick ratio, X16 cash flow ratio, and X21 returns on assets before tax, interest, and depreciation. The sequence of variables by degree of importance is X12, X16, X14, X02, X21 and X11.

**Table 3:** Selection results of decision tree CHAID

Variables	Variable importance
X12 (debt ratio)	0.2347
X16 (cash flow ratio)	0.2181
X14 (quick ratio)	0.2119
X02 (current assets ratio)	0.1337
X21 (returns on assets before tax, interest, and depreciation)	0.1282
X11 (operating expenses ratio)	0.0734

### Construction of the models and cross validation

This study applies SPSS Clementine for modeling purposes and uses CART, CHAID, BBN, SVM and ANN in order to construct models and evaluate the classification performance of the variables selected by the two algorithms’ DTs. After normalization of the selected variables, random sampling without repetition is conducted. The rigorous tenfold cross validation is also adopted in this study for testing classification accuracy.

### CART models

As shown in Table 4, the fraudulent financial statement detection accuracy and overall accuracy of the CART–CART model were the highest at 88.59 and 83.19 %, respectively. This study also discloses each model’s Type I and Type II errors, as shown in Table 5. Type I errors indicate fraudulent financial statements that have been mistakenly labeled as not being fraudulent. Type II errors indicate fraudulent financial statements which have been classified as fraudulent financial statements. Logically, a Type I error is a major mistake. Therefore, in addition to comparison with the accuracy of the testing group, the model’s Type I error rate is also considered. The CART–CART model has the lowest Type I error rate at 11.41 % and an overall error rate of 16.81 %.

**Table 4:** Detection accuracy of CART models—tenfold cross validation

Model	FFS (%)	Non-FFS (%)	Overall accuracy (%)
CART–CART	88.59	77.78	83.19
CART–CHAID	81.88	79.51	80.70
CART–BBN	77.18	73.22	75.20
CART–SVM	75.17	74.19	74.68
CART–ANN	75.84	74.16	75.00

**Table 5:** Type I error and Type II error of CART models

Model	Type I error rate (%)	Type II error rate (%)	Overall error rate (%)
CART–CART	11.41	22.22	16.81
CART–CHAID	18.12	20.49	19.30
CART–BBN	22.82	26.78	24.80
CART–SVM	24.83	25.81	25.32
CART–ANN	24.16	25.84	25.00

### CHAID models

As shown in Table 6, the fraudulent financial statement detection accuracy and overall accuracy of the CHAID–CART model are the highest at 92.69 and 87.97 %, respectively. Each model’s Type I and Type II errors are shown in Table 7. The CHAID–ANN model has the lowest Type I error rate at 7.31 %; the Type II error rate is 16.76 %, and the overall error rate is 12.03 %.

**Table 6:** Detection accuracy of CHAID models—tenfold cross validation

Model	FFS (%)	Non-FFS (%)	Overall accuracy (%)
CHAID–CART	92.69	83.24	87.97
CHAID–CHAID	79.19	71.37	75.28
CHAID–BBN	81.88	80.13	81.01
CHAID–SVM	79.87	78.23	79.05
CHAID–ANN	83.20	81.59	82.40

**Table 7:** Type I error and Type II error of CHAID models

Model	Type I error rate (%)	Type II error rate (%)	Overall error rate (%)
CHAID–CART	7.31	16.76	12.03
CHAID–CHAID	20.81	28.63	24.72
CHAID–BBN	18.12	19.87	18.99
CHAID–SVM	20.13	21.77	20.95
CHAID–ANN	16.80	18.41	17.60

### Statistical test

In terms of statistical tests, this study employs the t-test and the Wilcoxon rank-sum test. The t-test and the Wilcoxon rank-sum test of the various models, including CART–CART, CART–CHAID, CHAID–CHAID, CHAID–CART, CART–BBN, CART–SVM, CART–ANN, CHAID–BBN, CHAID–SVM, and CHAID–ANN, all reveal statistically significant levels (significant at P < 0.01), as shown in Table 8 (the t-test) and Table 9 (the Wilcoxon rank-sum test).

**Table 8:** The t-test of the models

Model	CART	CHAID	df	P value
CART	134.147***	155.612***	9	0.000
CHAID	190.738***	115.696***	9	0.000
BBN	117.247***	307.987***	9	0.000
SVM	117.391***	193.874***	9	0.000
ANN	169.994***	223.735***	9	0.000

* Significant at P < 0.1; ** significant at P < 0.05; and *** significant at P < 0.01

**Table 9:** The Wilcoxon rank-sum test of the models

Model	Mean rank		Z score	P value
	CART	CHAID		
CART	7.1	13.9	−2.5324	0.0057
CHAID	15.1	5.9	3.4395	0.0003
BBN	5.5	15.5	−3.7418	<0.0001
SVM	5.5	15.5	−3.7418	<0.0001
ANN	5.5	15.5	−3.7418	<0.0001

## Conclusions

A company’s financial statement is the key basis for all investor judgments, and is the last line of defense for investor interests. If management attempts to withhold information, even when independent CPAs, investment banking and securities analysts are involved, investors can experience significant losses. The most well-known scandal is the Enron bankruptcy case. Top management intentionally misled investors so as to profit by one billion USD. This caused bankruptcy for many investors and employees, and impacted the accounting and business community heavily.

The Enron case caused investors to lose confidence in financial statements and led to the establishment of the Sarbanes–Oxley Act (1992), which mandates that companies form auditing committees headed by independent directors. The Enron case resulted in the reform of accounting standards and the reconstruction of regulatory mechanisms.

In fact, unusual signs in financial statements often occur before the outbreak of a scandal. For example, signs of revenue situations, cash flow conditions and the ratio of liabilities and assets can all exist. Irrationalities can be found in financial statements from a few quarters to 1 year before the event. Fraudulent financial statements may look highly presentable, and many investors may be cheated. However, it is better to prevent fraud, protect investors from being cheated, and ensure that criminals are punished. Whether legal norms and supervision requirements are stringent enough and whether or not corporate governance can prevent intentional and deliberate acts of stealing company assets by manipulating financial statements are therefore issues that need to be addressed.

An increasing number of cases of fraudulent financial statements are able to damage companies and result in major losses for investors. People pay a heavy price to compensate for this damage. Therefore, establishing an effective fraudulent financial statement detection model is considerably important.

This study aims to provide a non-conventional analysis method by using multiple data mining techniques, including: the DT, BBN, SVM and ANN in order to construct a more accurate fraudulent financial statement detection model. In the first stage, this study applies the DTs of CART and CHAID to select the important variables. CART, CHAID, BBN, SVM and ANN are then combined in order to construct a classification model for comparison. According to the research results, the detection performance (overall accuracy) of the CHAID–CART model is the best at 87.97 % (the FFS detection accuracy is 92.69 %). It also has the lowest Type I error rate of 7.31 %. The sequences for overall accuracy are 83.19 % for the CART–CART model, 82.40 % for the CHAID–ANN model, 81.01 % for the CHAID–BNN model, 80.70 % for the CART–CHAID model, 79.05 % for the CHAID–SVM model, 75.28 % for the CHAID–CHAID model, 75.20 % for the CART–BNN model, 75.00 % for the CART–ANN model, and 74.68 % for the CART–SVM model.

Based on the empirical results of this study, the accuracy of the DT CHAID, combined with CART, in detecting fraudulent financial statements, is relatively high. It can therefore be used as a tool to help auditors in the detection of fraudulent financial statements. The research findings can provide a reference for investors, shareholders, company managers, credit rating institutions, auditors, CPAs (certified public accountants), securities analysts, financial regulatory authorities, and relevant academic institutions.

## References

[CR1] Arminger G, Enache D, Bonne T (1997). Analyzing credit risk data: a comparison of logistic discrimination classification tree analysis and feed forward networks. Comput Stat.

[CR2] Beasley M (1996). An empirical analysis of the relation between the board of director composition and financial statement fraud. Account Rev.

[CR100] Beasley MS, Carcello JV, Hermanson DR (1999) Fraudulent financial reporting 1987-1997: an analysis of U.S. public companies. The Committee of Sponsoring Organizations of the Treadway Commission (COSO), New York

[CR3] Beaver WH (1966). Financial ratios as predictors of failure. J Account Res.

[CR4] Bell T, Carcello J (2000). A decision aid for assessing the likelihood of fraudulent financial reporting. Audit A J Pract Theory.

[CR150] Breiman L, Friedman JH, Olshen RA, Stone CI (1984) Classification and regression trees. Wadsworth Publishing Co., Belmont

[CR5] Chen CH (2005). Application of grey forecast theory and logit equation in financial crisis warning model from the pre-event control viewpoint. Commer Manag Q.

[CR6] Chen G, Firth M, Gao DN, Rui OM (2006). Ownership structure, corporate governance, and fraud: evidence from China. J Corp Financ.

[CR7] Chiu CC, Lee TS, Chou YC, Lu CJ (2002). Application of integrated identification analysis and ANN in data mining. J Chin Inst Ind Eng.

[CR8] Coats PK, Fant LF (1993). A neural network approach to forecasting financial distress. J Bus Forecast.

[CR9] Elliot R, Willingham J (1980). Management fraud: detection and deterrence.

[CR10] Fanning K, Cogger K (1998). Neural network detection of management fraud using published financial data. Int J Intell Syst Account Financ Manag.

[CR11] Hansen JV, McDonald JB, Stice JD (1992). Artificial intelligence and generalized qualitative-response models: an empirical test on two audit decision-making domains. Decis Sci.

[CR12] Humpherys SL, Moffitt KC, Burns MB, Burgoon JK, Felix WF (2011). Identification of fraudulent financial statements using linguistic credibility analysis. Decis Support Syst.

[CR13] Kamarudin KA, Ismail WAW, Mustapha WAHW (2012). Aggressive financial reporting and corporate fraud. Procedia Soc Behav Sci.

[CR14] Kirkos S, Spathis C, Manolopoulos Y (2007). Data mining techniques for the detection of fraudulent financial statements. Expert Syst Appl.

[CR15] Koh HC (2004). Going concern prediction using data mining techniques. Manag Audit J.

[CR16] Kotsiantis S, Koumanakos E, Tzelepis D, Tampakas V (2006). Forecasting fraudulent financial statements using data miming. World Enformatika Soc.

[CR17] Larsson T, Patriksson M, Strömberg AB (1996). Conditional subgradient optimization—theory and applications. Eur J Oper Res.

[CR200] McCulloch WS, Pitts WH (1943). A logical calculus of the ideas immanent in nervous activity. Bull Math Biophys.

[CR18] Newton AC, Stewart GB, Diaz A, Golicher D, Pullin AS (2007). Bayesian belief networks as a tool for evidence-based conservation management. J Nat Conserv.

[CR19] Nguyen MN, Shi D, Quek C (2008). A nature inspired Ying–Yang approach for intelligent decision support in bank solvency analysis. Expert Syst Appl.

[CR20] Pai PF, Hsu MF, Wang MC (2011). A support vector machine-based model for detecting top management fraud. Knowl Based Syst.

[CR250] Pearl J (1986). Fusion, propagation, and structuring in belief networks. Artif Intell.

[CR21] Quinlan JR (1986a) C4.5: programs for machine learning. Morgan Kaufmann Publishers, Burlington

[CR22] Quinlan JR (1986b) C5.0: programs for machine learning. Morgan Kaufmann Publishers, Burlington

[CR300] Quinlan JR (1993) C4.5: Programs for machine learning. Morgan Kaufmann Publishers, Burlington

[CR23] Ravisankar P, Ravi V, Rao GR, Bose I (2011). Detection of financial statement fraud and feature selection using data mining techniques. Decis Support Syst.

[CR24] Rezaee Z (2005). Causes, consequences, and deterrence of financial statement fraud. Crit Perspect Account.

[CR25] Ribeiro CC, Minoux M, Penna MC (1989). An optimal column-generation-with-ranking algorithm for very large scale set partitioning problems in traffic assignment. Eur J Oper Res.

[CR26] Salehi M, Fard FZ (2013). Data mining approach to prediction of going concern using classification and regression tree (CART). Glob J Manag Bus Res.

[CR27] Seifert JW (2004). Data mining and the search for security: challenges for connecting the dots and databases. Gov Inf Q.

[CR28] Sharma VD (2004). Board of director characteristics, institutional ownership, and fraud: evidence from Australia. Audit A J Pract Theory.

[CR29] Shin KS, Lee TS, Kim HJ (2005). An application of support vector machines in bankruptcy prediction model. Expert Syst Appl.

[CR30] Spathis C, Doumpos M, Zopounidis C (2002). Detecting false financial statements: a comparative study using multicriteria analysis and multivariate statistical techniques. Eur Account Rev.

[CR31] Stamelos I, Angelis L, Dimou P, Sakellaris E (2003). On the use of bayesian belief networks for the prediction of software productivity. Inf Softw Technol.

[CR32] Summers SL, Sweeney JT (1998). Fraudulently misstated financial statements and insider trading: an empirical analysis. Account Rev.

[CR33] Tang A, Nicholson A, Jin Y, Han J (2007). Using bayesian belief networks for change impact analysis in architecture design. J Syst Softw.

[CR34] Uzun H, Szewczyk SH, Varma R (2004). Board composition and corporate fraud. Financ Anal J.

[CR35] Vapnik V (1995). The nature of statistical learning theory.

[CR350] Viaene S, Dedene G, Derrig R (2005). Auto claim fraud detection using Bayesian learning neural networks. Expert Syst Appl.

[CR36] Wells JT (1997) Occupational fraud and abuse. Obsidian Book Publishing, Nottingham

[CR37] Yeh CC, Chi DJ, Hsu MF (2010). A hybrid approach of DEA, rough set and support vector machines for business failure prediction. Expert Syst Appl.

[CR38] Zhou W, Kapoor G (2011). Detecting evolutionary financial statement fraud. Decis Support Syst.

